# Supplementary material for: Super-broadband on-chip continuous spectral translation unlocking coherent optical communications beyond conventional telecom bands
Source: Nat Commun. 2022 Jul 16;13:4139. doi: 10.1038/s41467-022-31884-2 (PMC9288461; doi:10.1038/s41467-022-31884-2)
Supplement: Supplementary file 1 — Supplementary Information [file 41467_2022_31884_MOESM1_ESM.pdf]

Super-broadband On-chip Continuous Spectral Translation Unlocking Coherent Optical Communications Beyond Conventional Telecom Bands

Supplementary Information

Deming Kong[1,*], Yong Liu[1], Zhengqi Ren[2], Yongmin Jung[2], Chanju Kim[1], Yong Chen[2], Natalie V Wheeler[2], Marco N Petrovich[2], Minhao Pu[1], Kresten Yvind[1], Michael Galili[1], Leif K Oxenløwe[1], David J Richardson[2], and Hao Hu[1,*]

[1]DTU Fotonik, Technical University of Denmark, DK-2800, Kgs. Lyngby, Denmark

[2]Optoelectronics Research Centre, University of Southampton, Southampton, SO17 1BJ, UK

Supplementary Note 1 – High-order phase matching

The conversion efficiency (CE) of degenerate four-wave mixing (FWM) can be determined by CE $= \gamma^2 P_{\mathrm{p}}^2 L_{\mathrm{eff}}^2 \eta$, where γ is the nonlinear coefficient, and P_{p} is the optical power of the pump. L_{eff} is the effective length of the nonlinear waveguide and $L_{\mathrm{eff}} = \left(1 - e^{-\alpha L}\right)/\alpha$, where α is the loss per unit length and L is the length of the waveguide. The normalised CE is defined as[1]:

$$\eta = \frac{\alpha^2}{\alpha^2 + \Delta\beta^2}\left(1 + \frac{4e^{-\alpha L}\sin^2\left(\Delta\beta L/2\right)}{\left(1 - e^{-\alpha L}\right)^2}\right) \tag{1}$$

where $\Delta\beta$ is the phase mismatch per unit length of the waveguide. The phase mismatch is a sum of linear and power-dependent nonlinear terms:

$$\Delta\beta = \Delta\beta_{\mathrm{linear}} + 2\gamma P_{\mathrm{p}} \tag{2}$$

where $\Delta\beta_{\text{linear}} = 2\beta_{\text{p}} - \beta_{\text{s}} - \beta_{\text{i}}$, and β_{p}, β_{s}, β_{i} are the propagation constant of the pump, signal, and idler, respectively. Here we assume that the optical power of the signal and the idler is significantly smaller than the pump power. Consequently, the nonlinear phase mismatch originates from self-phase modulation of the pump. The overall phase mismatch term should be minimized for a large CE. If the pump is in the small power region the contribution of nonlinear phase mismatch can be ignored. The linear phase mismatch can be expanded at the pump frequency and simplified to

$$\Delta\beta_{\text{linear}} = \beta_2 \Delta\omega^2 + \frac{1}{12}\beta_4 \Delta\omega^4 \tag{3}$$

where the first and second terms come from the second and fourth-order dispersion. To minimize the phase mismatch, i.e., $\Delta\beta_{\text{linear}} = 0$, we have

$$\Delta\omega = \pm\sqrt{-12\left(\beta_2/\beta_4\right)} \tag{4}$$

This means that two phase matching points exist in addition to the one where the signal and the pump have the same frequency (i.e., $\Delta\omega = 0$). The conversion bands associated with these phase matching points are defined as high-order phase matching bands. As these phase matching points are a function of the pump frequency, a large continuous conversion band can be achieved by detuning the pump from the zero-dispersion wavelength (ZDW) and exploiting the high-order phase matching bands.

Supplementary Note 2 – Advantages of the 2-µm band

The 2-µm band is currently a leading contender among all the new wavelength bands due to the combination of several emerging technologies. Firstly, the thulium-doped fibre amplifier (TDFA) offers more than 240-nm bandwidth in the 2-µm band with high gain and low noise figure[2,3]. Secondly, hollow-core fibre (HCF), e.g., using photonic bandgap or anti-resonant effects, enables data transmission in the 2-µm band. HCF could potentially achieve a loss of the order of 0.1 dB/km[4], surpassing the best conventional silica fibres[5]. Photonic bandgap fibres have been made with >150-nm bandwidth in the 2-µm band[6] and antiresonant fibres with 700-nm bandwidth covering both the conventional C band and the 2-µm band[7]. HCF also features low latency transmission at >99.7% of the speed of light[8], and ultralow nonlinearity[9]. Just as low-loss standard single-mode fibre (SSMF) and the erbium-doped fibre amplifier (EDFA) have boosted the optical fibre telecom

industry, HCF and the TDFA could ultimately spawn novel 2-µm-band optical communication systems, allowing signals to be relayed over long distances with low cost and low energy consumption. In addition, the 2-µm band is attractive for silicon photonics due to the reduced two-photon absorption[10,11], allowing advanced on-chip functionalities.

Supplementary Note 3 – Measured spectral flatness of the translation

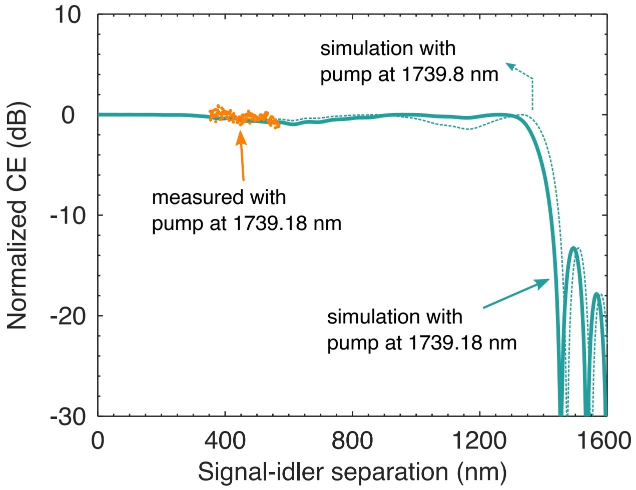

Supplementary Figure 1 Measured flatness of the translation band. The measurement is done with a CW signal that is swept from 1500 nm to 1580 nm, while the pump is a CW beam from a DFB laser at 1739.18 nm. The continuous curve shows the simulation results with a pump wavelength of 1739.18 nm. The dashed curve shows the simulation results with the pump at the optimum wavelength of 1739.8 nm.

We have measured the flatness of the translation band with a continuous-wave (CW) signal that is swept from 1500 nm to 1580 nm with an interval of 0.5 nm. The results are shown in Supplementary Figure 1 with scatter points, along with a comparison to the simulation results. Note that, in this measurement, the pump originates from a distributed feedback (DFB) laser working at 1739.18 nm. The solid and dash-dotted curves show simulation results for the translation band when the pump is tuned to 1739.18 nm and the optimum wavelength of 1739.8 nm, respectively. It can be seen that an offset from the optimum pump wavelength would result in a slightly decreased conversion bandwidth, but the conversion bandwidth is still sufficient for the spectral translation from the C band to the 2-µm band and vice versa. In the 2-µm-band transmission experiment, the pump laser is thermally tuned to 1739.74 nm, which is very close to the optimum pump

wavelength. Nevertheless, the measured results of the flatness of the translation band match well with the simulation and a maximum fluctuation of the conversion efficiency of < 2 dB is achieved.

Supplementary Note 4 – Spectral translation of an optical frequency comb

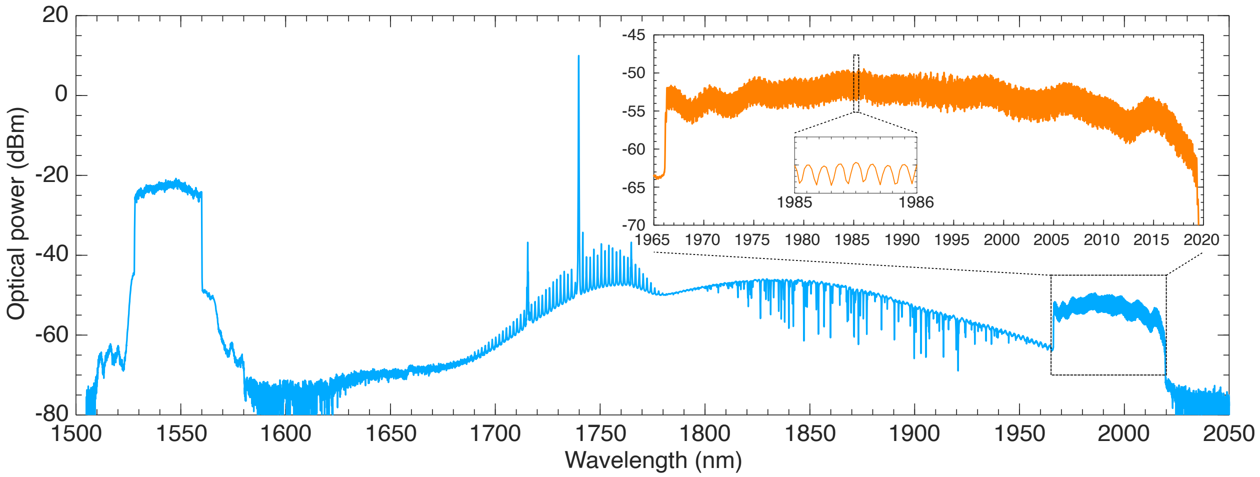

Supplementary Figure 2 Spectral translation of a 10-GHz spaced optical frequency comb covering most of the telecom C band (1528.0 nm to 1560.0 nm) to the 2-µm band. The result demonstrates that the AlGaAsOI nanowaveguide based spectral translator has a flat conversion band and is capable of the spectral translation of a wideband multi-channel signal.

In addition to the transmission experiment, we have also performed spectral translation of an optical frequency comb (OFC) from the C band to the 2-µm band to explore the potential of our spectral translators. The OFC originates from a seed 1.5-ps Gaussian pulse train out of a mode-locked laser with a centre wavelength of 1543.5 nm, a repetition rate of 10 GHz, and a 20-dB spectral width of 6.4 nm. The seed OFC from the laser is spectrally broadened through on self-phase modulation in a piece of 400-m length of dispersion-flattened highly nonlinear fibre (DF-HNLF) ($\gamma = 10.5\ \mathrm{W}^{-1}\,\mathrm{km}^{-1}$, $\beta_2 = 0.567\ \mathrm{ps}^2/\mathrm{km}$, $\beta_3 = 0.008\ \mathrm{ps}^3/\mathrm{km}$)[12] with a launch power of 26.5 dBm, resulting in a broadened OFC with a 10-dB bandwidth of 40 nm spanning from 1524.0 nm to 1563.9 nm. Then, the OFC is spectrally shaped by a wavelength-selective switch to a flat-top spectrum spanning from 1528.0 nm to 1560.0 nm. After amplification, the OFC is aligned in polarization with the pump and finally launched into the spectral translator to be converted to the 2-µm band. Supplementary Figure 2 gives the output spectra from the spectral translator, with the zoomed-in spectrum showing the converted 2-µm-band OFC. The shape of the OFC is well preserved after the spectral translator,

confirming a flat conversion bandwidth across the telecom C band. The large conversion bandwidth shown in the simulation results and experimental measurements demonstrates that the spectral translator could be a very promising solution to bridge between many wavelength bands.

Supplementary Note 5 – Latency measurement for the HCF

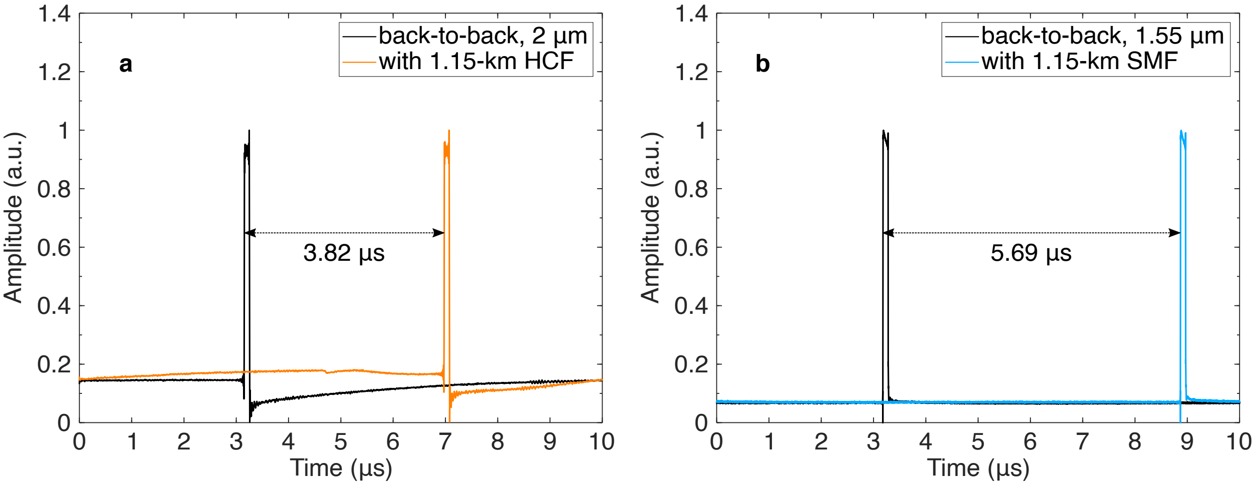

Supplementary Figure 3 Measured pulse propagation delays. **a**, Pulse propagation delay of the 2-µm signal through the 1.15-km HCF. **b**, Pulse propagation delay of the C-band signal through the 1.15-km SSMF.

As well as the wide transmission band for 2-µm signals, the HCF has an overwhelming advantage in terms of latency (pulse propagation delay). We have measured the latency of the ∼1.15-km HCF for the 2-µm-band signal, and have made a similar measurement of the latency of a C-band signal transmitted through a 1.15-km length of SSMF. We use a pulse train generated from an arbitrary waveform generator with a repetition rate of 100-kHz and a duty cycle of 1%. The pulse train is modulated into the optical domain by Mach-Zehnder modulators (Fujitsu FTM7938EZ for C band and EOSpace AZ-0S5-20-PFA-PFA-2000-LV for 2-µm band). A photodiode (Discovery Semiconductor DSC2-50S) with a 3-dB bandwidth of 12 GHz and a spectral wavelength range from 1200 nm to 2200 nm is used to detect the pulse trains before and after transmission. The measured results for the 2-µm-band and C-band pulse propagation delays are shown in Supplementary Figure 3a and Supplementary Figure 3b, respectively. Despite some inaccuracy in the exact length of the HCF and SSMF, the pulse propagation delay is reduced from 5.69 µs to 3.82 µs by shifting from the C band with SSMF to the 2-µm band in the HCF, showing a latency reduction of 33%, as anticipated.

References

[1] Tkach, R., Chraplyvy, A., Forghieri, F., Gnauck, A. & Derosier, R. Four-photon mixing and high-speed WDM systems. *Journal of Lightwave Technology* **13**, 841–849 (1995).

[2] Li, Z. *et al.* Diode-pumped wideband thulium-doped fiber amplifiers for optical communications in the 1800 – 2050 nm window. *Opt. Express* **21**, 26450–26455 (2013).

[3] Li, Z. *et al.* Thulium-doped fiber amplifier for optical communications at 2 Âµm. *Optics Express* **21**, 9289–9297 (2013).

[4] Roberts, P. J. *et al.* Ultimate low loss of hollow-core photonic crystal fibres. *Opt. Express* **13**, 236–244 (2005).

[5] Chigusa, Y. Ultra-low-loss (0.1484 db/km) pure silica core fibre and extension of transmission distance. *Electronics Letters* **38**, 1168–1169(1) (2002).

[6] Petrovich, M. N. *et al.* Demonstration of amplified data transmission at 2 Âµm in a low-loss wide bandwidth hollow core photonic bandgap fiber. *Optics Express* **21**, 28559 (2013).

[7] Sakr, H. *et al.* Ultrawide bandwidth hollow core fiber for interband short reach data transmission. In *2019 Optical Fiber Communications Conference and Exhibition (OFC)*, 1–3 (2019).

[8] Slavík, R. *et al.* 1.45 Tbit/s, low latency data transmission through a 19-cell hollow core photonic band gap fibre. In *European Conference and Exhibition on Optical Communication*, Mo.2.F.2 (Optical Society of America, 2012).

[9] Liu, Z. *et al.* Nonlinearity-free coherent transmission in hollow-core antiresonant fiber. *Journal of Lightwave Technology* **37**, 909–916 (2019).

[10] Liu, X., Osgood, R. M., Vlasov, Y. A. & Green, W. M. J. Mid-infrared optical parametric amplifier using silicon nanophotonic waveguides. *Nature Photonics* **4**, 557–560 (2010).

[11] Zlatanovic, S. *et al.* Mid-infrared wavelength conversion in silicon waveguides using ultracompact telecom-band-derived pump source. *Nature Photonics* **4**, 561–564 (2010).

[12] Galili, M. *et al.* Generation and detection of 2.56 Tbit/s OTDM data using DPSK and polarisation multiplexing. In *Optical Fiber Communication Conference*, OThV2 (OSA, 2010).
